# The changing faces of cholangitis

**DOI:** 10.12688/f1000research.8745.1

**Published:** 2016-06-17

**Authors:** Sum P. Lee, Joseph R. Roberts, Rahul Kuver

**Affiliations:** 1 Department of Medicine, Division of Gastroenterology , University of Washington School of Medicine, Seattle, WA, USA

**Keywords:** cholangitis, primary sclerosing cholangitis, primary biliary cholangitis, IgG4-associated sclerosing cholangitis, cholangitis research

## Abstract

A variety of diseases are included under the umbrella term ‘cholangitis’, including hepatobiliary diseases with an autoimmune pathogenesis (such as primary sclerosing cholangitis, primary biliary cholangitis, and IgG4-associated sclerosing cholangitis) and disease processes associated with intraductal stones and infectious etiologies (such as ascending bacterial cholangitis, recurrent pyogenic cholangitis, and liver fluke-associated cholangitis). Recent advances in the pathophysiologic bases of these disorders, particularly with respect to the autoimmune variety, are allowing improved diagnosis and prognostication as well as providing the opportunity to refine and re-imagine treatment modalities. The aim of this review is to highlight selected advances in cholangitis research that point to novel insights into the pathophysiology, diagnosis, and treatment of this diverse array of disorders.

## Introduction

The term ‘cholangitis’ taken at face value means inflammation in the biliary system. This simple terminology is deceptive, however, as it encompasses a wide variety of diseases. These include liver fluke-associated cholangitis, ascending bacterial cholangitis associated with choledocholithiasis, recurrent pyogenic cholangitis, IgG4-associated cholangitis (IAC, also referred to as IgG4-related sclerosing cholangitis [ISC]), and primary sclerosing cholangitis (PSC). Recently, the designation for primary biliary cirrhosis (PBC) was changed to primary biliary cholangitis (conveniently also abbreviated to PBC) ^1^, bringing yet another autoimmune disorder that affects the hepatobiliary system under the cholangitis umbrella. The term cholangiopathy has also been used to categorize several of these disorders, as the disease locus is the cholangiocyte or biliary epithelial cell ^2^. The aim of this review is to summarize and put into perspective selected advances reported within the last 3 years that provide novel insights in our understanding of the pathophysiology, diagnosis, and treatment of these disorders.

## Pathophysiology: new faces in the crowd

### Mechanisms of immune-mediated injury: roles for natural killer T cells and the CD14 receptor

PSC and PBC are autoimmune disorders, and a central tenet in the pathophysiology of these disorders is the complex interactions between cholangiocytes and cells in the innate and adaptive immune systems. Recent studies point to a central role for natural killer T (NKT) cells in the pathophysiology of these disorders ^3^. The ability of cholangiocytes to express antigen-presenting molecules is established, but they were not previously known to activate NKT cells, a cell type abundant in the murine liver and also found to a lesser degree in the human liver. NKT cells respond to antigens presented with the major histocompatibility complex (MHC) class I-like molecule CD1d. Cultured human cholangiocytes and cholangiocarcinoma cells presented exogenous CD1d-restricted antigens to invariant NKT cell clones. Cd1d expression was downregulated in the biliary epithelium of patients with late PSC and PBC compared to healthy controls. This demonstrates that NKT cells can be activated by biliary epithelium and thereby regulate inflammation, which may be pathophysiologically relevant for PSC and PBC ^4^. NKT cells also regulate T cell responses in PBC both during the initiation of disease as well as in the chronic phase ^5^. A high ratio of NKT cells to cholangiocytes led to cytotoxicity of autologous cholangiocytes. At a low NKT/cholangiocyte ratio, cholangiocytes were not lysed, but interferon gamma (IFNγ) production was induced. This facilitated the expression of MHC class I and II molecules on cholangiocytes and afforded protection from lysis upon subsequent exposure to autoreactive T cells. Therefore, NKT cell-mediated innate immune responses are critical at the initial stage of PBC and also maintain the chronic cytopathic effect of autoantigen-specific T cells ^5^. Invariant NKT cell-deficient mice have decreased portal inflammation and reduced anti-mitochondrial antibody responses ^6^. These studies point to an important role for the innate immune system in general, and NKT cells in particular, in disease initiation and progression in both PBC and PSC.

CD14 receptor signaling is another component of the innate immune system, and this receptor is constitutively expressed in the majority of innate immune cells. A recent report implicates this pathway in the pathogenesis of intrahepatic biliary strictures. Bile duct strictures are a hallmark of PSC and are a major cause of clinical consequences, causing biliary obstruction with ascending cholangitis and setting the stage for cholangiocarcinoma. CD14 receptor signaling was linked to dominant stricture formation in PSC patients in association with a specific genotype ^7^.

### The gut microbiome: a nexus for the primary sclerosing cholangitis/inflammatory bowel disease interface

The gut microbiome is a key component in the maintenance of health, and alterations in this meta-organ are increasingly being linked to the pathophysiologic basis for a wide variety of diseases ^8^. Such alterations are particularly relevant for autoimmune conditions, including PSC. Approximately 75% of patients with PSC also have inflammatory bowel disease (IBD), predominantly ulcerative colitis (UC). This intersection of IBD with PSC has been the focus of studies to elucidate pathophysiologic mechanisms that could shed light on both diseases. Potential mechanisms for this close association between PSC and IBD include alterations in gut flora leading to changes in immune responses that could affect biliary epithelia and alterations in bile acid composition and signaling (e.g. via farnesoid X receptor [FXR], TGR5, and/or FGF19-mediated pathways) by gut microbial populations that could have downstream effects on biliary epithelia.

Murine studies point to a role for the gut microbiome in biliary epithelial homeostasis. In the mdr2 (-/-) model of PSC, absence of the gut microbiota exacerbated hepatobiliary disease as shown by increased cholangiocyte senescence and more severe phenotypic features of PSC. Ursodeoxycholic acid (UDCA) abrogated cholangiocyte senescence ^9, 10^. These results demonstrate that the gut microbiota confers protection against injury to the biliary epithelium.

Recent reports are beginning to shed light on microbial diversity with respect to patients with PSC (with and without IBD) in comparison to healthy controls. In one recent study, the gut microbial profile in patients with PSC was distinct from UC patients without biliary disease and healthy controls but similar in PSC patients with IBD ^11^. PSC patients had reduced bacterial diversity compared to healthy controls. The *Veillonella* genus, which is also associated with other chronic inflammatory and fibrotic conditions, was more abundant in patients with PSC ^11^. In another study, the mucosa-associated microbiota of patients with PSC was characterized by increased numbers of bacteria belonging to the *Blautia* and *Barnesiellaceae* genera and by major shifts in operational taxa units within the *Clostridiales* order ^12^. In contrast, another group reported no strong PSC-specific microbial associations in UC patients ^13^. While these studies are limited by small sample size and methodologic shortcomings, we anticipate future studies that will expand our understanding of the role of the gut microbiome in PSC and IBD. Two recent reviews highlight the current understanding of the gut microbiota with respect to hepatobiliary health and disease ^14, 15^.

### Circulating exosomes in primary biliary cholangitis: regulation of co-stimulatory molecules

Exosomes are secreted nanoparticles originating from endocytic vesicles that mediate cell-to-cell communication. Exosomes isolated from PBC patients and healthy controls showed effects on co-stimulatory molecule expression and cytokine production in mononuclear cells. There were differences in microRNA expression in circulating exosomes in patients with PBC compared to exosomes from healthy controls. These exosomes altered co-stimulatory molecule expression on antigen-presenting cells. These results suggest that aberrant exosomes in PBC selectively induce the expression of co-stimulatory molecules in different subsets of antigen-presenting cells and could contribute to the pathogenesis of PBC ^16^.

## Diagnosis: effacing uncertainty

### Defining the primary sclerosing cholangitis/inflammatory bowel disease interface

Defining the relationship between PSC and IBD has implications that affect the clinical management of patients who carry either diagnosis. New evidence suggests that PSC associated with IBD is a distinct clinical entity compared to IBD without PSC. Disease-specific patterns at shared genetic loci showed strong co-morbidity between PSC and IBD, suggesting that PSC/IBD is a disease that is unique from IBD ^17^. Patients with IBD and PSC have a markedly higher risk for the development of colorectal neoplasia than patients with IBD alone. UC rather than Crohn’s disease (CD) is associated with this higher risk in PSC patients ^18^. The prevalence of PSC with CD is rare and carries a more benign prognosis as compared to patients with PSC with UC or without IBD. Approximately 25% of patients with concomitant PSC/CD had small duct PSC. In large duct PSC/CD, the liver disease was less aggressive and had a more favorable outcome ^19^. Prolonged duration of IBD is associated with an increased risk of cholangiocarcinoma in patients with PSC/IBD, and colectomy does not modify this risk ^20^. Screening for biliary malignancy with ERCP and brush cytology showed that even in asymptomatic PSC patients, 43% had advanced disease and 7% presented with suspicious or malignant brush cytology at first endoscopic retrograde cholangiography ^21^. These studies highlight the ongoing efforts to delineate the interface between PSC and IBD.

### IgG4-related sclerosing cholangitis: diagnostic challenges

Cholangitis is one of the common organ manifestations of IgG4-related disease; approximately 60% of patients with this systemic condition have changes consistent with sclerosing cholangitis in the proximal and/or distal bile ducts. ISC can result in hilar and even intrahepatic bile duct stenoses that can be difficult to differentiate from PSC and cholangiocarcinoma ^22^. To further complicate matters, evidence is emerging that there is shifting of the diagnostic boundaries among ISC, biliary tract malignancy, and PSC ^23^. Differential diagnosis between ISC and cholangiocarcinoma can be facilitated by a multi-modal diagnostic approach that includes the use of tumor markers, serum IgG4 levels, and other organ involvement. Differentiation between ISC and PSC can be improved by IgG4 ^+^ BCR clones and IgG4/IgG RNA ratio ^24^. Response to steroid therapy can also help delineate these conditions in difficult cases ^25^. However, a case report of extensive metastatic cholangiocarcinoma that was misdiagnosed as ISC illustrates these diagnostic limitations ^26^. To further cloud the picture, PSC patients can have elevated IgG4 levels in serum, which is linked to specific human leukocyte antigen (HLA) haplotypes. Patients with the highest levels of IgG4 had significantly lower frequency of the strongest PSC risk factor, HLA-B*08, than patients without elevated IgG4. HLA genotype therefore might affect the serum concentration of IgG4, and increased IgG4 might be a marker of a distinct phenotype of PSC ^27^. ISC and autoimmune pancreatitis have also been associated with extra-pancreatic organ failure and malignancy, requiring careful surveillance of these patients ^28^. These studies highlight the ongoing clinical challenges regarding the diagnosis and management of patients when clinical features of ISC, PSC, and cholangiocarcinoma overlap.

### Biomarker development: microRNAs and more

Serum and biliary biomarkers for early identification of PSC and cholangiocarcinoma are the subject of ongoing studies ^29^. MicroRNAs have shown promise, with miR-1281, miR-126, miR-26a, miR-30b, and miR-122 showing significant differences between patients with PSC and patients with cholangiocarcinoma. In addition, these microRNAs were significantly lower in healthy individuals ^30^. In bile, levels of miR-412, miR-640, miR-157, and miR-189 were significantly different between patients with PSC and patients with PSC/cholangiocarcinoma ^31^. Another potential biomarker that is elevated in PSC is Pr3-ANCA; it can distinguish PSC from autoimmune hepatitis and PBC ^32^. PSC can also be distinguished by elevated levels of biomarkers of apoptosis as opposed to biomarkers of necrosis ^33^. Similarly, a panel of serum microRNAs was reported to be a more sensitive and specific biomarker for PBC than alkaline phosphatase or anti-nuclear antibodies ^34^. Serum cell death biomarkers have also been found to predict liver fibrosis and poor prognosis in patients with PBC ^35^.

Traditional serum biomarkers continue to be refined. For example, normalization of alkaline phosphatase is a biomarker of improved survival in PSC and decreased likelihood of requiring orthotopic liver transplantation ^36^. Reduction in alkaline phosphatase is also associated with longer survival in PSC independent of the presence of dominant strictures ^37^. The serum biomarker CA 19-9 is associated with inflammation but not biliary obstruction in PSC patients ^38^. Serum and urine bile acid and carnitine profiles may also be potential biomarkers in PBC ^39^. The enhanced liver fibrosis score, which is a serum-based test of hyaluronic acid, TIMP-1, and propeptide of type III procollagen, has been shown to predict transplant-free survival in PSC ^40^. Serum alkaline phosphatase and bilirubin levels are surrogate markers of outcomes in PBC ^41^. Therefore, traditional and newer biomarkers continue to be developed and refined for use as tools for earlier and more specific diagnosis, as well as for helping with prognostication.

### Endoscopic technology: refining diagnostic accuracy

Endoscopic technology continues to improve, allowing greater accuracy in the diagnosis of indeterminate biliary strictures, a challenge in the evaluation of dominant strictures in PSC. Cholangioscopy, using more sophisticated technological platforms, continues to evolve ^42^. Cholangioscopes, confocal endoscopy, endoscopic ultrasound, and magnetic resonance-based imaging techniques provide ever more detailed information regarding strictures and other pathologic lesions in the biliary system. An example is the use of digital single operator cholangioscopy, which showed 85% sensitivity and 100% specificity for the diagnosis of malignancy arising within biliary strictures ^43^.

## New treatments: facing a bright future

### Primary biliary cholangitis: moving beyond ursodeoxycholic acid

For decades, the mainstay of treatment for PBC has been UDCA, which is non-toxic and widely available and has proven efficacy. However, up to 40% of patients with PBC will have an inadequate response to UDCA. FXR is a nuclear hormone receptor that is central to bile acid homeostasis in the hepatobiliary-gut axis. FXR activation has been associated with anti-cholestatic, anti-inflammatory, and anti-fibrotic effects. Obeticholic acid (OCA) is a potent FXR ligand that is being studied for the treatment of a variety of disorders. A double-blind study to assess the efficacy of OCA in PBC patients was recently reported; 165 patients on a stable dose of UDCA and with elevated alkaline phosphatase levels and no other immunosuppression or immunomodulatory therapy were randomized to escalating doses of OCA or placebo for 3 months. There was a decrease in alkaline phosphatase levels, with the majority of the effect seen after 1 month, suggesting that OCA may be a useful adjunct therapy to patients with PBC on UDCA with persistent biochemical abnormalities ^44^. A dose-dependent side effect of OCA is pruritus, which is a common symptom in PBC patients. It is important to be cautious and introspective when considering OCA use. A long-term benefit has yet to be demonstrated. In addition, adverse serum lipid profiles and outcome may also severely limit its use.

### Novel therapies for primary biliary cholangitis and primary sclerosing cholangitis: promising but not yet proven

Several comprehensive reviews summarize the plethora of promising new therapies that are being tested in patients with PBC ^45– 47^. These novel approaches to treatment are based on insights into the cellular and molecular mechanisms involved in all stages of PBC, from the initiating and early immunologically mediated cholangiocyte injury to the damaging and disease-sustaining effects of cholestasis, leading ultimately to fibrosis and the development of cirrhosis. Therapies at various points in development include budesonide, fibrates, antivirals, B cell depletion with the anti-CD20 monoclonal antibody rituximab, anti-interleukin 12/interleukin 23 molecules such as ustekinumab, FGF-19 analogues, apical sodium-dependent bile acid transporter (ASBT) inhibitors, and TGR5 agonists, among others ^47^. This wide array of treatment modalities, many of which are based on novel insights into the physiology of bile acid signaling pathways, nuclear hormone receptor signaling, and immunologic targets, are a testament to the advances in understanding of the cellular and molecular basis of PBC that have been decades in the making. Results of these studies are eagerly awaited. Proof of efficacy of one or more of these agents could dramatically alter the clinical management of patients with PBC who do not respond to UDCA.

A similarly impressive array of novel therapeutics is under investigation for the medical management of PSC. Unlike PBC, the efficacy of UDCA remains unproven, with studies showing improvement in liver biochemistry profiles but no survival benefit. Furthermore, higher doses of UDCA are associated with adverse outcomes (reviewed in 48, with references therein). Due to this gap, there is great interest in developing effective therapies for this disease. Pre-clinical and clinical studies are ongoing and were recently reviewed ^49^. Promising agents include nuclear hormone receptor agonists such as OCA for FXR, fibrates for PPARalpha, curcumin for PPARgamma, vitamin D for the vitamin D receptor, and all-trans retinoic acid for RAR/RXR. Another promising agent is the side-chain shortened derivative of UDCA, 24-norursodeoxycholic acid (norUDCA) ^48^. Finally, agonists for TGR5 and for FGF-19 as well as inhibitors of ASBT are also candidates for drug development ^48^. These drugs will change the management and outlook of PSC if shown to be effective in clinical trials.

## Summary

This review highlights the many ways in which our understanding of the pathophysiology, diagnosis, and management of the various diseases that are collectively labeled as cholangitis are evolving. We have chosen to highlight selected recently published advances that demonstrate novel insights, the majority of which involve the cholangitides with an autoimmune pathogenesis. The field of cholangitis studies is entering an unprecedented era of new advances that promise to translate into more effective prevention and management of this pathophysiologically fascinating and clinically challenging family of disorders. From novel insights regarding the role of the innate and adaptive immune systems to the not-unrelated role of the gut microbiome, our understanding of how these diseases develop and progress is expanding. Biomarker development with microRNAs and technological advances in endoscopy and imaging promise to provide earlier and more accurate diagnoses. In the therapeutic realm, the pipeline is full, and all stakeholders—patients and their families; basic, translational, and clinical investigators; and clinicians, among others—should feel cautiously optimistic about more effective treatments and improved outcomes.
